# Comparative chemical array screening for p38γ/δ MAPK inhibitors using a single gatekeeper residue difference between p38α/β and p38γ/δ

**DOI:** 10.1038/srep29881

**Published:** 2016-07-19

**Authors:** Yasumitsu Kondoh, Kaori Honda, Sayoko Hiranuma, Teruo Hayashi, Takeshi Shimizu, Nobumoto Watanabe, Hiroyuki Osada

**Affiliations:** 1 Antibiotics Laboratory, RIKEN, Saitama 351-0198, Japan; 2 Chemical Biology Research Group, RIKEN Center for Sustainable Resource Science, Saitama 351-0198, Japan; 3 Bio-Active Compounds Discovery Research Unit, RIKEN Center for Sustainable Resource Science, Saitama 351-0198, Japan

## Abstract

Mammalian p38 mitogen activated protein kinases (MAPKs) are responsive to a variety of cellular stresses. The development of specific pyridinyl imidazole inhibitors has permitted the characterization of the p38 MAPK isoform p38α, which is expressed in most cell types, whereas the physiological roles of p38γ and p38δ are poorly understood. In this study, we report an approach for identifying selective inhibitors against p38γ and p38δ by focusing on the difference in gatekeeper residues between p38α/β and p38γ/δ. Using GST-fused p38α wild type and T106M mutant constructs, wherein the p38α gatekeeper residue (Thr-106) was substituted by the p38γ/δ-type (Met), we performed comparative chemical array screening to identify specific binders of the mutant and identified SU-002 bound to p38αT106M specifically. SU-002 was found to inhibit p38αT106M but not p38α kinase activity in *in vitro* kinase assays. SU-005, the analog of SU-002, had inhibitory effects against the kinase activity of p38γ and p38δ *in vitro* but not p38α. In addition, SU-005 inhibited both p38γ and p38δ auto-phosphorylation in HeLa and HEK293T cells. These results demonstrate that the comparative chemical array screening approach is a powerful technique to explore specific inhibitors for mutant proteins with even single amino-acid substitutions in a high-throughput manner.

Mammalian p38 mitogen-activated protein kinases (MAPKs) are responsive to a variety of extracellular stress stimuli^1^,^2^. There are four p38 MAPK isoforms in mammals: α, β, γ, and δ^3^,^4^,^5^,^6^,^7^,^8^,^9^. Among these, p38α, which is expressed in most cell types, is the best characterized. The p38 MAPK isoforms are divided into two distinct subsets based on their similarity. p38α and p38β are 75% identical in their amino-acid sequence, whereas p38γ and p38δ are only 62% and 61% identical to p38α, respectively^10^ but are more identical (~70%) to each other. Although p38α and p38β are inhibited by the pyridinyl imidazole inhibitors SB203580 and SB202190 (Fig. S1) in *in vitro* and *in vivo* assays, these drugs do not inhibit p38γ and p38δ^9^,^11^,^12^,^13^. To understand the exact functions of each p38 family member such as their different roles in various cell and/or tissue contexts, the development of specific inhibitors against the latter isoforms have been long awaited^2^. Furthermore, the lack of specific p38γ and/or p38δ inhibitors has delayed the identification of their *in vivo* substrates and understanding of their physiological roles. p38γ and p38δ knock-out mice, which are viable and have no apparent phenotype^14^, have provided important insights into the function of p38γ and p38δ *in vivo*. Recently, it was reported that p38γ and/or p38δ were involved in diseases such as inflammatory arthritis and diabetes^15^,^16^. In the collagen-induced arthritis model, both p38γ and p38δ deficiency markedly reduced arthritis severity with lower levels of cytokine production and pathogenic anti-collagen antibody responses than those in wild type mice^15^. Therefore, p38γ and p38δ are crucial regulators of inflammatory joint destruction and represent potential therapeutic targets in rheumatoid arthritis. In addition, p38δ deficiency was associated with improved glucose tolerance because of enhanced insulin secretion from pancreatic β cells^16^. p38δ deletion induced apparent activation of protein kinase D as a pivotal regulator of stimulated insulin exocytosis and provided protection against oxidative stress-mediated β cell failure^16^. Thus, p38δ also has a pivotal role in the regulation of insulin secretory capacity and survival of pancreatic β cells.

The diaryl urea compound BIRB796 (Fig. S1) inhibits all p38 isoforms at high concentrations *in vitro* and in cells but inhibits only p38α and p38β at low concentrations^13^. Thus, BIRB796 can be used to identify the physiological roles of these p38 isoforms by using varying concentrations of this compound alongside the p38α/β specific inhibitor SB203580^13^. p38δ inhibition attenuates IL13-induced airway mucus production in inflammatory airway diseases through a signaling pathway from chloride channel calcium-activated 1 (CLCA1) to p38δ^17^. BIRB796 at high concentration markedly inhibited the production of secretory mucin MUC5AC mRNA and protein, the major macromolecular constituent of airway mucus, in airway epithelial cells whereas SB203580 did not^17^. Alevy *et al*. developed more potent p38δ inhibitors, BIRB796 analogs (Fig. S1) with submicromolar potency, by using structure-based drug design^17^. However, these compounds also inhibited p38α activity with nanomolar potency, and therefore do not act as specific inhibitors against p38δ.

Examination of the crystal structure of p38α with SB203580 revealed that the drug inserted into the ATP-binding pocket of p38α and that three residues, Thr-106, His-107, and Leu-108, which lie at the back of the ATP-binding pocket, are important for pyridinyl imidazole inhibitor binding^18^. The fluoro-phenyl ring of SB203580 binds in a hydrophobic pocket at the back of the active site through van der Waals contacts^19^. Thr-106, the so-called “gatekeeper”, comes into contact with the inhibitor. Its substitution to a bulky residue such as Met, which p38γ and p38δ possess at the Thr-106 equivalent position in the ATP-binding pocket, causes loss of binding in p38α and p38β, resulting in drug insensitivity^11^,^18^. Specifically, the bulky Met at the Thr-106 equivalent position fills the hydrophobic pocket and interferes with the fluoro-phenyl ring^20^. Conversely substitution of the gatekeeper residue Met to Thr in p38γ and p38δ makes them sensitive to SB203580^11^. Thus, the gatekeeper residue contributes to p38 pyridinyl imidazole inhibitor selectivity.

Here, we report an alternative approach for identifying selective inhibitors against p38γ and p38δ by focusing on the difference of the gatekeeper residue between p38α/β and p38γ/δ. We generated GST-fused p38α wild type and p38αT106M mutant constructs, and identified compounds that specifically bound to the p38αT106M mutant by comparative screening using chemical arrays^21^,^22^ wherein 27,013 library compounds from the RIKEN NPDepo were immobilized. Compounds binding to p38αT106M but not to wild type p38α are more likely to discriminate the difference in the gatekeeper residue because p38αT106M only differs with respect to the wild type Thr-106 substituted with Met. This alternative approach could be applicable to primary screening for selective inhibitors by focusing on the difference of a single residue in a protein of interest in a rapid, low-cost manner.

## Results

### Screening Strategy of Compounds Specifically Binding to the p38αT106M Mutant (“Hit Compounds”)

**Figure 1:**
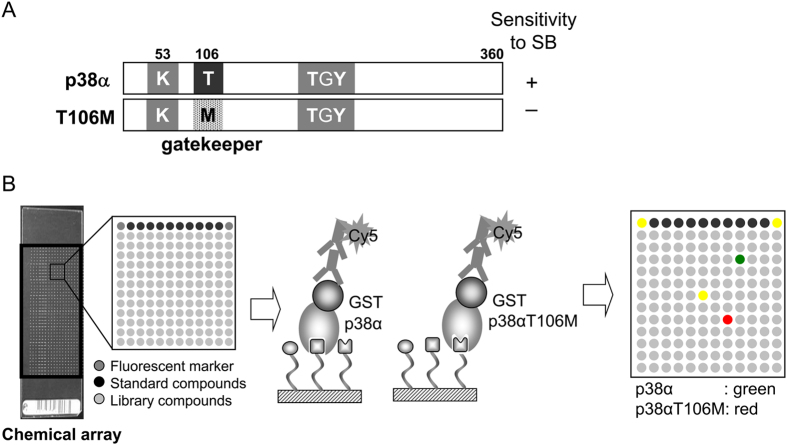
Schemes of p38α and p38αT106M and their use in comparative chemical array screening. **(A**) p38α and p38αT106M. The p38αT106M mutant contains the gatekeeper residue substitution Thr to Met and is insensitive to SB203580 (SB). (**B**) The chemical array had 16 or 24 blocks in duplicate and each block contained 144 library compounds, 10 standard compounds, and 2 fluorescent markers. The chemical arrays were treated with GST-p38α wild type or GST-p38αT106M mutant followed by anti-GST antibody and Cy5-labeled secondary antibody for immunostaining. Fluorescence images of chemical arrays probed with GST-p38α or GST-p38αT106M were labeled green or red, respectively. After the two arrays were merged, specific binders of GST-p38αT106M were detected as red spots, while specific binders of GST-p38α were green. Compounds binding to both proteins were detected as yellow spots.

The gatekeeper residue is the major determinant for inhibition of p38s by SB203580 and contributes to drug selectivity in p38 isoforms^11^. The kinase activities of p38α and p38β, whose gatekeepers are Thr-106, are inhibited by SB203580. In contrast, p38γ and p38δ possess Met at the Thr-106 equivalent position in the ATP-binding pocket and are insensitive to SB203580 from the associated lack of drug binding. Similarly, the p38αT106M mutant with Met substituted for Thr-106 is also insensitive to SB203580 (Fig. 1A)^11^,^23^. To identify inhibitors specific to p38γ/δ, the discovery of p38αT106M specific binders by comparative chemical array screening using p38αT106M mutant and p38α wild type proteins (Fig. 1B) might be informative. In particular, such binders might also bind to the ATP-binding pocket of p38γ and p38δ isoforms through discriminating the gatekeeper difference. Furthermore, this approach excludes compounds binding to both the p38αT106M mutant and wild type at sites common to both proteins, resulting in the exclusion of compounds binding with other sites than the ATP-binding pockets with differing gatekeeper residues.

### Identification of the Hit Compound SU-002 as a p38αT106M Specific Binder

**Figure 2:**
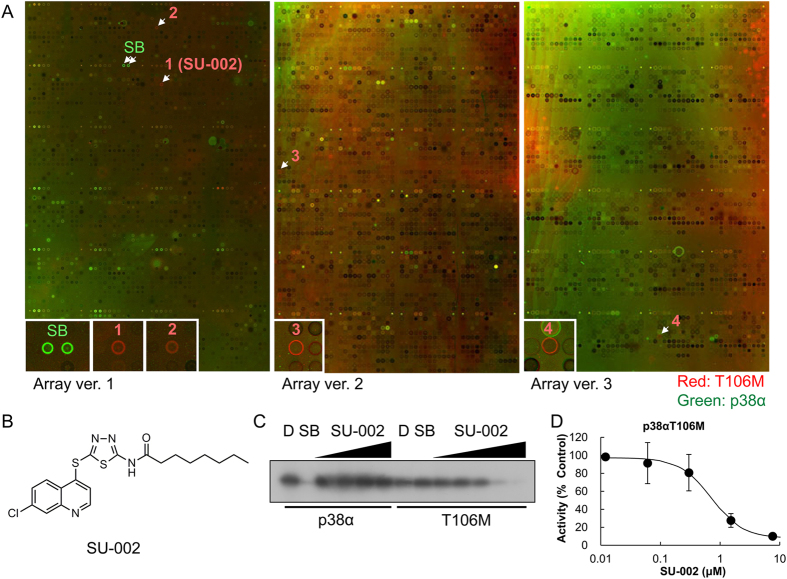
Screening of specific binders of the p38αT106M mutant. (**A**) Fluorescence images of the merged chemical arrays probed with GST-p38αT106M (red) and GST-p38α (green). Specific binders of GST-p38αT106M or GST-p38α are represented as red or green spots, respectively. SB203580 (SB) spots are green, illustrating its specific binding of GST-p38α. On the other hand, spots for compounds **1**–**4** are red, illustrating their specific binding to GST-p38αT106M. The chemical array version including compounds **1**–**4** are indicated below each images (Array version: 1, 2, and 3). (**B**) Chemical structure of SU-002. (**C**) *In vitro* kinase assay of GST-p38α and GST-p38αT106M. ATF2 was used as a substrate for the kinase assay. D: DMSO, SB: SB203580. DMSO was used as a negative control. The concentration of SB203580 was 1.0 μM; the concentrations of SU-002 were 0.06, 0.3, 1.5, and 7.6 μM in p38α (from left) and 0.012, 0.06, 0.3, 1.5, and 7.6 μM in p38αT106M (from left). (**D**) Concentration-response curve of p38αT106M by SU-002. Results are presented as %kinase activity relative to that in control incubations where the compound was omitted (means of triplicate determinations, N = 3). Error bar represents the standard error.

We generated GST-fused p38αT106M mutant and p38α wild type recombinant proteins and performed screening of their potential binders using photo-cross-linked chemical arrays whereupon 27,013 compounds of the RIKEN NPDepo were immobilized. The chemical arrays treated with GST-p38α or GST-p38αT106M were labeled in green or red, respectively; images of the merged arrays are shown in Fig. 2A. The SB203580 spots were labeled green, demonstrating that SB203580 specifically bound to the p38α wild type protein on the chemical array (Fig. 2A left panel). Notably, the fluorescence intensity (Ι) scores of the SB203580 spots in p38α were very high, but those for p38αT106M were negligible (Table S1). The ΔI scores, which represent the intensity difference between p38α and p38αT106M, were larger than 10 (Table S1). This result demonstrates that SB203580 molecules immobilized on the chemical array selectively bind to the p38α wild type protein by discriminating the difference of a single residue between p38α wild type and p38αT106M mutant proteins.

Next, we identified p38αT106M specific binders by comparing the data of p38αT106M with those of p38α and found 4 compounds among the 27,013 compounds. Spots for compounds **1**–**4**, indicated in Fig. 2A, appeared in red, demonstrating that they bound to p38αT106M on the chemical array but not to p38α wild type. The ΔΙ score of compound **1** (SU-002, Fig. 2B) was highest among the 4 compounds (Table S2). We then examined the inhibitory activity of SU-002 against GST-p38α and GST-p38αT106M by *in vitro* kinase assay. The results demonstrated that SU-002 inhibited the kinase activity of p38αT106M in a dose-dependent manner but this inhibition was not seen in the case of p38α (Fig. 2C,D). In contrast, the other 3 compounds did not inhibit the kinase activities of either p38α or p38αT106M at the tested concentration. These results indicated that the comparative chemical array screening approach enabled us to discover compounds that specifically bound to p38α or the p38αT106M mutant by discriminating a single amino acid residue difference between p38α and p38αT106M.

### Inhibition of p38αT106M, p38γ, and p38δ by SU-002 Derivatives

**Figure 3:**
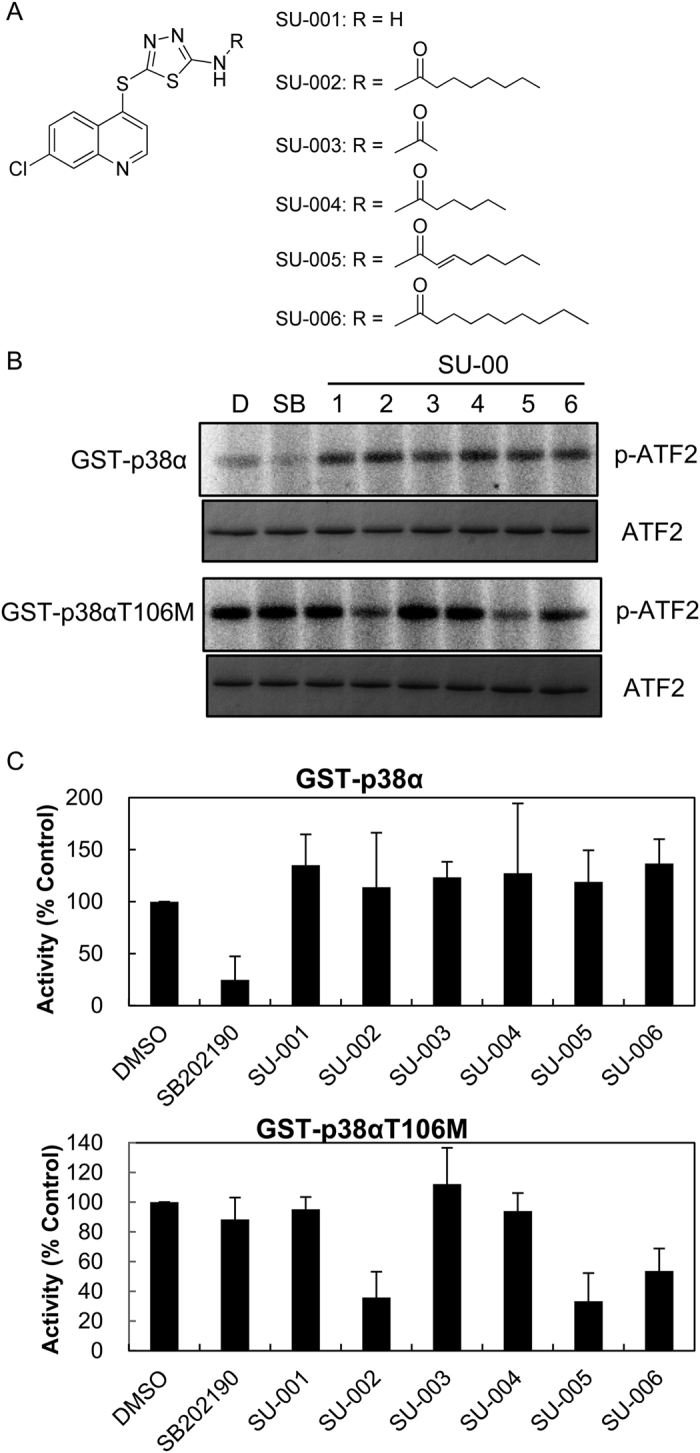
Inhibitory effects of SU-002 and its derivatives against p38αT106M in an *in vitro* kinase assay. (**A**) Chemical structure of the SU-002 and its derivatives (SU-001, 3, 4, 5, 6). (**B**) Inhibitory activities of SU-002 and its derivatives in an *in vitro* kinase assay. ATF2 was used as a substrate for the kinase assay. The upper panel represents phosphorylated ATF2 and the lower panel represents the ATF2 loading control. D: DMSO, SB: SB202190, SU-00: SU001-SU006 (final concentration 1 μM). DMSO was used as a negative control. (**C**) Percentage of residual kinase activity of GST-p38α and GST-p38αT106M at 1 μM of SU-002 or its derivatives relative to control (DMSO). Values are the means ± SD represented by vertical bars (N = 2).

**Figure 4:**
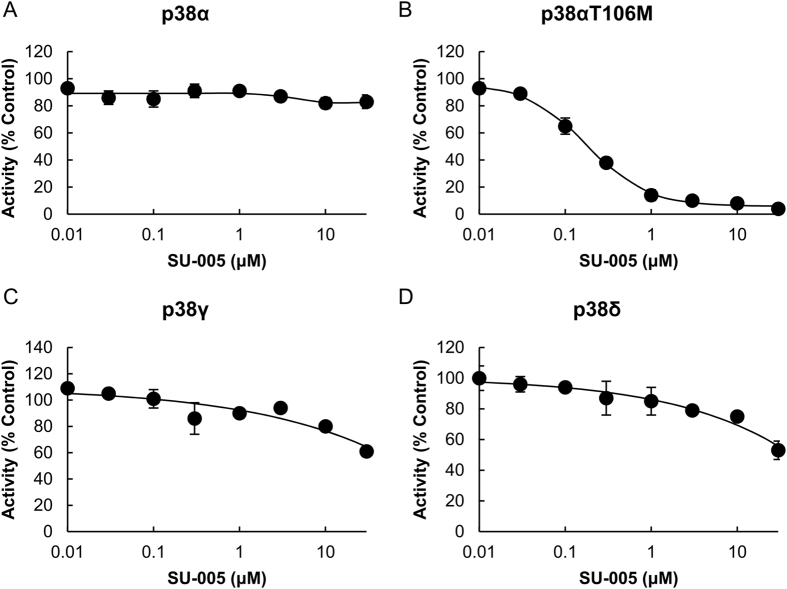
Concentration-response curves of four pre-activated p38 isoforms by SU-005. (**A**) p38α. (**B**) p38αT106M. (**C**) p38γ. (**D**) p38δ. Results are presented as %kinase activity relative to that in control incubations where the compound was omitted (means of duplicate determinations, N = 2). The error bar indicates SD. The ATP concentration was the Km value (90 μM in p38α; 70 μM in p38αT106M; 15 μM in p38γ and p38δ).

**Figure 5:**
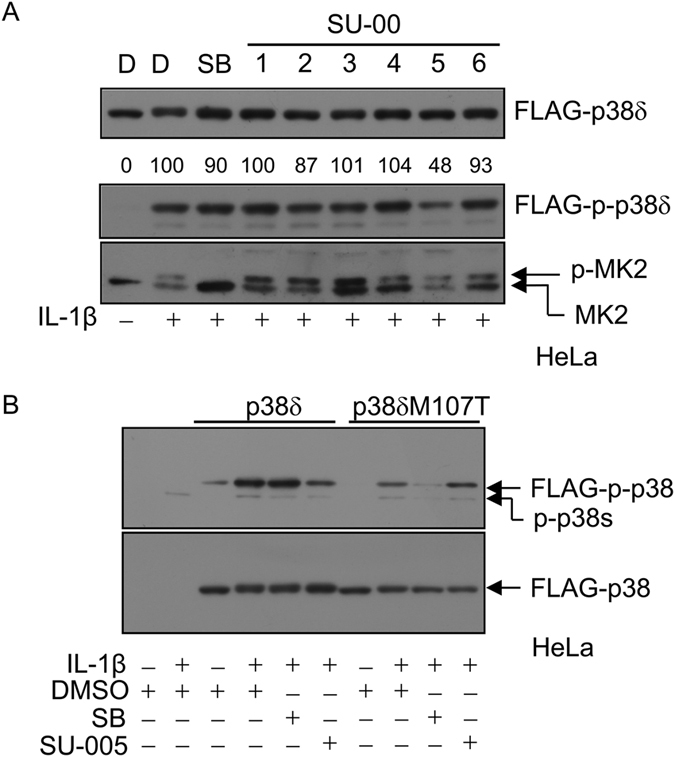
Inhibitory effects of SU-005 against the phosphorylation of over-expressed p38δ. (**A**) SU-005 decreased the phosphorylation of over-expressed p38δ but did not affect endogenous p38α activity in HeLa cells. MK2: MAPKAPK2. p-MK2: phosphorylated MAPKAPK2. D: DMSO, SB: SB202190, SU-00: SU001-SU006 (final concentration 10 μM). (**B**) A gatekeeper amino acid substitution in p38δ abrogated its sensitivity to SU-005 and conferred sensitivity to SB202190.

SU-002 derivatives (SU-001, 003, 004, 005, and 006, shown in Fig. 3A) were synthesized and the inhibitory activities of SU-002 and its derivatives against p38αT106M were tested. As shown in Fig. 3B,C, SU-002 and SU-005 clearly impaired p38αT106M kinase activities in *in vitro* kinase assays whereas SB202190 inhibited p38α activity. We then conducted quantitative kinase assay analyses including IC_50_ using the KinaseProfiler service of Eurofins Pharma Discovery Services. MBP was used as a substrate for all tested p38 isoforms: p38α, p38αT106M, p38γ, and p38δ. All p38 isoforms used were activated using a constitutively active mutant of MKK6. SU-005 exhibited the most potent inhibitory activity against p38αT106M (IC_50_: 184 nM) (Fig. 4, S2 and Table S3). SU-005 demonstrated moderate inhibitory activities against p38γ and p38δ dose-dependently; however, we were unable to measure IC_50_ owing to the insolubility of SU-005 at concentrations of more than 30 μM. Next, we examined their effects in HeLa cells. As shown in Fig. 5A, SU-005 impaired the phosphorylation of over-expressed FLAG-tagged p38δ more effectively than did SU-002 (middle panel). On the other hand, the inhibition of phosphorylation of MK2 (a p38α specific substrate) was only observed with SB202190 treatment (Fig. 5A, bottom panel). Together, these data suggest that these compounds can penetrate into cells and are therefore applicable for intracellular functional studies of p38 isoforms other than those sensitive to SB202190/203580. To verify the specificity of SU-005 for p38δ, a FLAG-tagged p38δM107T was constructed and tested in a similar manner. We found that SU-005 did not inhibit p38δM107T whereas SB202190 inhibited p38δM107T auto-phosphorylation, as SU-005 inhibited that of p38δ (Fig. 5B). These data suggested that SU-005 might target the gatekeeper residue Met such as within p38δΜ107.

### Inhibition of Endogenous p38γ Phosphorylation by SU-002 and SU-005

**Figure 6:**
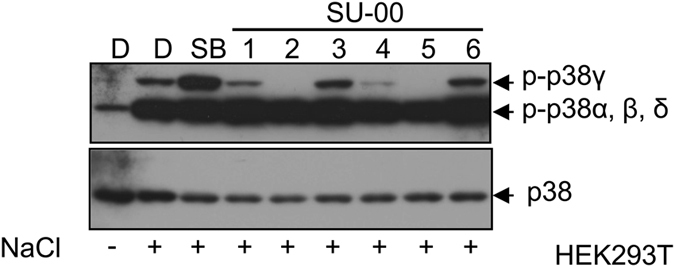
Endogenous p38γ auto-phosphorylation inhibition by SU-002 and SU-005 in HEK293T cells. D: DMSO, SB: SB202190, SU-00: SU001-SU006 (final concentration 10 μM).

In the course of the study, we noticed the presence of an anti-phospho p38 antibody reactive signal that migrated between the major p38s (p38α, possibly including p38β and δ) and FLAG-tagged p38γ only when we used HEK293T cells. The profile of phosphorylation always coincided with that of Flag-p38γ. We therefore asked whether this signal originated from endogenous p38γ, and verified this suggestion by using an anti-p38γ antibody. Subsequently, endogenous p38γ phosphorylation was therefore used as the indicator for compound inhibitory activity. As shown in Fig. 6, SU-002 and SU-005 again demonstrated similarly potent inhibitory activities against endogenous p38γ phosphorylation. Together, the data indicate that SU-005 might serve as a promising p38γ and/or p38δ specific inhibitor applicable not only for research but also for therapeutic purposes.

## Discussion

Chemical arrays have been powerful tools for the high throughput screening of binders for a protein of interest. However, binders identified by chemical array screening often include compounds without desired properties such as inhibitory or activation activity toward a protein of interest, because the binding sites of the compounds on the protein molecule cannot be limited to specific sites in chemical array screening. To solve this issue, we conceived of a comparative chemical array screening approach to identify compounds binding to a focused site of protein using both wild type and mutant proteins containing a substituted residue on a focused site (Fig. 1). The identification of compounds binding to wild type but not to mutant proteins or vice versa enables us to discover compounds binding to each type of protein at a specified site. For this purpose, chemical arrays require the ability to robustly detect the interaction between a compound and specific protein binding site. The p38α MAPK inhibitor SB203580 was shown to bind to the p38α wild type but not to the p38αT106M mutant, which is insensitive to this drug, on a chemical array, suggesting that our chemical array system can detect the interaction between SB203580 and the ATP-binding site of p38α (Fig. 2A and Table S1).

As a proof-of-concept of the comparative chemical array screening approach, we performed screening for compounds that selectively bound to the p38αT106M mutant, in which the gatekeeper residue Thr-106 in the ATP-binding pocket is substituted with a bulky residue Met that is present within p38γ and p38δ at the Thr-106 equivalent position. Among the 27,013 screened compounds, 4 were selected as compounds that potentially bound to p38αΤ106Μ but not to the p38α wild type protein. The overall screening hit rate was very low because this approach excluded compounds that bound to both the p38αT106M mutant and the wild-type at sites common to both proteins. One compound, SU-002, with a 2-(7-chloroquinolin-4-yl)sulfanyl-1,3,4-thiadiazole (CQT) structure was identified as an inhibitor of the p38αT106M mutant in subsequent *in vitro* kinase assays (Fig. 2). Furthermore, SU-005 impaired the kinase activities of p38γ and p38δ but not of p38α both in *in vitro* and in cells (Figs 4, 5, 6). Although the inhibitory activities of SU-005 against pre-activated p38γ and p38δ were weak (Fig. 4), SU-005 effectively inhibited the auto-phosphorylation of p38γ and p38δ (Figs 5 and 6). Furthermore, whereas SU-002 and SU-005 exhibit similar inhibitory activity potencies against endogenous p38γ phosphorylation, SU-005 has a more potent inhibitory activity against FLAG-tagged p38δ phosphorylation than does SU-002 (Figs 5 and 6). However, the CQT structure was not sufficient for the inhibitory activity, because SU-001 did not inhibit the kinase activities of p38αT106M (Fig. 3). It was observed that the attachment of an alkyl amide to the CQT structure in addition to its carbon chain length was important for inhibitory activity. SU-003 or SU-004, which possessed short alkyl chains, acetamide or hexanamide, respectively, showed no or weak inhibitory activity against auto-phosphorylation of the endogenous p38γ, whereas SU-002 and SU-005, which possessed long alkyl chains, octanamide or oct-2-enamide, respectively, inhibited its kinase activity in cells (Fig. 6). SU-006 possessed a longer alkyl chain, decanamide, than SU-002 and SU-005 but showed no inhibitory activity against the p38γ kinase. These data suggest that an octanamide or oct-2-enamide attached to CQT might be required to interact with the Met-type gatekeeper in ATP-binding pockets. Furthermore, our hypothesis is supported by the data that SU-005 had no inhibitory activity against p38δM107T mutants (Fig. 5).

A fluoro-phenyl group of SB203580 fills a hydrophobic pocket located deep within its ATP-binding site (Fig. S3A)^24^. The gatekeeper residue Thr106 of p38α is located within the deep hydrophobic pocket. Small residues such as threonine at position 106 allow the fluoro-phenyl ring to fill the hydrophobic pocket but larger residues such as methionine, as found in p38γ, reduce the hydrophobic pocket volume and block binding of the fluoro-phenyl ring (Fig. S3B)^20^. Our identified CQT inhibitors SU-002 or SU-005 require octanamide or oct-2-enamide for inhibitory activity against p38γ. From this result, we speculate that these attached amides might fit into the shallow hydrophobic pocket formed by Met109 in p38γ. To confirm this speculation, structural determination of p38γ in complex with SU-002 or SU-005 would be required.

Our results suggest that SU-005 might be useful as a bioprobe to elucidate the physiological function of p38γ and/or p38δ. Furthermore, studies in mouse models suggest that these molecules are crucial regulators of inflammatory joint destruction and also mediate insulin secretion from pancreatic β cells with concomitant changes in glucose tolerance^15^,^16^. Thus, our identified p38γ and/or p38δ inhibitors SU-005 might have the potential to be used as therapeutic agents for rheumatoid arthritis and diabetes.

In summary, our comparative chemical array screening approach enabled us to discover the binders interacting at a focused ATP-binding pocket of a p38αT106M mutant that mimicked the gatekeeper of p38γ or p38δ in a high throughput manner. This approach could be applicable to other proteins as well. For example, substitutions of the gatekeeper residues of some kinases are known to confer resistance to kinase inhibitors. The clinical efficacies of epidermal growth factor receptor (EGFR) kinase inhibitors such as gefitinib and erlotinib are limited in EGFR-mutant non-small-cell lung cancer in which EGFR bears the gatekeeper substitution T790M^25^,^26^. Futhermore, resistance of the mutation of BCL-ABL, which bears the gatekeeper substitution T315I, to imatinib has been observed in chronic myeloid leukemia^27^,^28^. Agents specifically inhibiting such drug-resistant kinases but not their wild type counterparts are generally clinically more potent and less toxic than those that concurrently inhibit both wild type and mutant kinases. Our comparative chemical array screening approach might be applicable to screen such agents for drug-resistant kinases bearing gatekeeper substitutions in a high throughput manner.

## Methods

### Materials

Chemicals were purchased from either Wako Pure Chemical Industries or Sigma-Aldrich. All commercially available chemicals for chemical synthesis were used without further purification. Lipidure BL-1002 was purchased from NOF Corporation.

Recombinant human p38α and p38αT106M mutant proteins tagged with glutathione-*S*-transferase at the N-terminus were purified via their affinities to glutathione Sepharose^29^. Anti-GST antibody (rabbit IgG fraction) was purchased from Invitrogen. Goat anti-rabbit IgG-Cy5 conjugate was purchased from Millipore. Recombinant human interleukin-1β was purchased from Pepro Tech EC Ltd. MBP and anti-FLAG M2 antibody were purchased from Sigma-Aldrich Japan K.K. Anti-phospho-p38 MAPK (T180/Y182) (D3F9) and anti-MAPKAPK-2 antibodies were from Cell Signaling Technologies. Anti-p38α antibody was prepared as described previously^30^.

### Plasmid Construction

For the expression of human p38 isoform proteins, first p38γ (from Kazusa DNA Res. Inst. Japan) and p38δ (from a HepG2 cell line cDNA library) were cloned into the ClaI/ApaI site of pcDNA3Flag-hp38^31^. A mutant with a single amino acid substitution, pcDNA3Flag-hp38δM107T, was constructed using the previously described method^32^. Primers for amino acid substitution were designed specifically for this thermal cycling mutagenesis. To express these proteins in *Escherichia coli*, each ClaI/ApaI fragment from the above plasmids was cloned into pRSET-CA^29^. All coding region nucleotide sequences of the newly constructed plasmids were confirmed by DNA sequencing.

### Cell Culture

HeLa and HEK293T cells (RIKEN Cell Bank, Tsukuba, Japan) were cultured in Dulbecco’s modified Eagle’s medium (Invitrogen) supplemented with 10% (v/v) fetal bovine serum (FBS) (JRH Biosciences), 50 U/mL penicillin, and 50 μg/mL streptomycin (Gibco) at 37 °C with 5% CO_2_.

### Synthesis of 4a (SU-002) and 4b (SU-005)

Synthesis of **4a** (SU-002) and **4b** (SU-005) is shown in Scheme S1.

### Synthesis of Thiadiazole Derivative 2

NaH (60% dispersion in mineral oil, 48 mg, 1.2 mmol) was added to a solution of 5–amino–1,3,4–thiadiazolo-2–thiol (147 mg, 1.1 mmol) in DMF (8 mL) at room temperature under a nitrogen atmosphere. After stirring for 20 min, 4,7–dichloroquinoline (218 mg, 1.1 mmol) was added and the reaction mixture was heated at 80 °C for 18 h. The reaction mixture was then cooled to room temperature and 40 mL water was added. The precipitate was separated by filtration, washed with cold water several times and with diethyl ether to afford product **2** (SU–001, 260 mg, 80.0%).

**2** (SU-001): TLC (CHCl_3_:MeOH, 10:1 v/v): Rf = 0.31; ^1^H NMR (500 MHz, CDCl_3_): δ 8.79 (d, J = 4.6 Hz, 1H), 8.20 (d, J = 9.1 Hz, 1H), 8.14 (d, J = 2.3 Hz, 1H), 7.82 (brs, 2H), 7.77 (dd, J = 9.1, 2.3 Hz, 1H), 7.23 (d, J = 4.6 Hz, 1H); ^13^C NMR (125 MHz, CDCl_3_): δ 173.2, 151.4, 147.9, 144.2, 141.8, 135.0, 128.4, 128.1, 125.4, 123.9, 119.8; HRMS (ESI) m/z: [M+H]^+^ calcd. for C_11_H_8_ClN_4_S_2_, 294.9879; found, 294.9879.

### Synthesis of Thiadiazole Derivatives 4a and 4b

To a stirred solution of **2** (0.2 mmol) in THF (5 mL), octanoic acid **3a** or *trans*-2-octenoic acid **3b** (0.25 mmol) and EDC hydrochloride (0.3 mmol) were added at room temperature. The reaction mixture was stirred at 50 °C for 12 h. After cooling down to room temperature, the mixture was diluted with CHCl_3_, washed with 1N NaHCO_3_ solution, H_2_O, and brine, and then dried over Na_2_SO_4_. After evaporation of the solvent, the residue was purified by preparative TLC (CHCl_3_: MeOH = 20: 1) to provide **4a** (SU-002, 87.1%) or **4b** (SU-005, 87.3%) as a colorless solid, respectively.

**4a (**SU-002): TLC (CHCl_3_:MeOH, 10:1 v/v): Rf = 0.48; ^1^H NMR (500 MHz, CDCl_3_): δ 8.80 (d, J = 4.6 Hz, 1H), 8.24 (d, J = 9.2 Hz, 1H), 8.16 (d, J = 1.7 Hz, 1H), 7.60 (dd, J = 9.2, 1.7 Hz, 1H), 7.40 (d, J = 4.6 Hz, 1H), 2.64 (t, J = 7.4 Hz, 2H), 1.73 (m, 2H), 1.24–1.34 (m, 8H), 0.84 (t, J = 6.9 Hz, 3H); ^13^C NMR (125 MHz, CDCl_3_): δ 171.9, 162.8, 154.7, 150.8, 148.9, 141.8, 136.5, 129.3, 128.7, 125.4 (x 2), 122.8, 36.2, 31.6, 29.0, 28.9, 25.1, 22.6, 14.0; HRMS (ESI) m/z: [M+H]^+^ calcd. for C_19_H_22_ClN_4_OS_2_, 421.0924; found, 421.0915.

**4b (**SU–005): TLC (CHCl_3_:MeOH, 10:1 v/v): Rf = 0.48; ^1^H NMR (500 MHz, CDCl_3_): δ 8.80 (d, J = 4.6 Hz, 1H), 8.24 (d, J = 9.2 Hz, 1H), 8.16 (d, J = 1.7 Hz, 1H), 7.60 (dd, J = 9.2, 1.7 Hz, 1H), 7.42 (d, J = 4.6 Hz, 1H), 7.25 (dt, J = 15.5, 6.9 Hz, 1H), 6.46 (d, J = 15.5 Hz, 1H), 2.26 (dt, J = 7.4, 6.9 Hz, 2H), 1.51 (tt, J = 7.4, 6.9 Hz, 2H), 1.27 (m, 4H), 0.83 (t, J = 6.9 Hz, 3H); ^13^C NMR (125 MHz, CDCl_3_): 164.0, 163.8, 154.6, 151.9, 150.8, 148.8, 141.9, 136.4, 129.2, 128.7, 125.3 (x 2), 122.6, 121.2, 32.5, 31.3, 27.4, 22.4, 13.9; HRMS (ESI) m/z: [M+H]^+^ calcd. for C_19_H_20_ClN_4_OS_2_, 419.0767; found, 419.0763.

### Preparation of Photo-Cross-Linked Chemical Arrays

Photo-cross-linked chemical arrays were prepared by the previously described method^33^ utilizing a photoaffinity proline linker that had both PEG and a polyproline helix as spacers^33^. We used 27,013 library compounds from the RIKEN NPDepo for the chemical arrays and arrayed these compounds onto ten separate photoaffinity-linker-coated glass slides.

### Comparative Screening using GST-p38α and GST-p38αT106M

The chemical array slides were blocked with binding buffer (1% skimmed milk, 0.2% Lipidure BL-1002, 10 mM Tris/HCl, 150 mM NaCl, and 0.05% Tween-20, pH 8.0) for 1 h at room temperature. The slides were then washed with TBS-T buffer (10 mM Tris/HCl, 150 mM NaCl, 0.05% Tween-20, pH 8.0) three times for 5 min each and centrifuged (480 × *g*, 1 min) to dry. The slides were then incubated with 1.4 μM GST-p38α or GST-p38αT106M in binding buffer at 30 °C for 1 h. After washing with TBS-T buffer, the slides were incubated with an anti-GST antibody (30 μg/mL) in binding buffer at 30 °C for 1 h. This incubation was followed by another wash step and incubation with a secondary antibody (goat anti-rabbit IgG, Cy5 conjugate, 50 μg/mL in binding buffer) at 30 °C for 1 h. After the final wash step and spin dry, the slides were scanned at 635 nm on a GenePix 4300A microarray scanner (Molecular Devices). The fluorescence intensity (I) of each compound spot was quantified with GenePix 5.0 software with local background correction. Then, the difference (ΔΙ) of the fluorescence intensities between the GST-p38αT106M and GST-p38α-treated arrays was calculated by subtracting I_p38α_ from I_T106M_ (ΔI = I_T106M_ - I_p38α_) for each spot. The mean and standard deviation (SD) of ΔI for all spots on the array were also calculated. The ΔΙ score of each spot was calculated using the following formula; ΔΙ score = (ΔI - mean)/SD. The compounds with a ΔΙ score larger than 2 were determined to be hit compounds for GST-p38αT106M. The fluorescence images of GST-p38αT106M- or GST-p38α-treated arrays were labeled red or green respectively using Adobe Photoshop image analysis software. Images of the GST-p38αT106M- and GST-p38α-treated arrays were then merged using Photoshop.

### Kinase Assay

Bacterially produced GST-p38α and GST-p38αT106M were used for the kinase assay. GST-ATF2 (1–109)^34^ was used as a substrate. The assay conditions used have been previously described^35^. GST-p38 isoforms (0.5 μg) were mixed with a test compound in DMSO (final concentration: 1 μM) and kinase buffer (25 mM Hepes, pH 7.3, 20 mM β-glycerophosphate, 20 mM MgCl_2_, 2 mM DTT, and 0.1 mM sodium orthovanadate). DMSO (1 μL) was used instead of compound as a negative control. Then, the mixture was combined with the solution containing 1 μg substrate protein, 185 kBq [γ-^32^P]ATP and 20 μΜ ATP to generate a 15 μL reaction mixture and incubated for 30 min at 30 °C. The reaction was terminated by adding 7.5 μL of 3 × SDS sample buffer and boiled for 5 min. Phosphorylated substrate protein was then separated by 12.5% SDS- polyacrylamide gel electrophoresis and visualized by autoradiography.

Quantitative kinase assay analyses including IC_50_ were conducted using the KinaseProfiler service of Eurofins Pharma Discovery Services UK Limited.

### Transfection and Western Blotting

For the transfection assay, HeLa cells were seeded into each well of a 24-well plate at 0.5 × 10^5^ cells per well. The next day, the expression plasmid was transfected with Effectene (Qiagen) according to the manufacturer’s instructions. These cells were grown in DMEM supplemented with 10% FBS for 24 h followed by stimulation as indicated in Fig. 3C,D. Then, SU derivative or SB202190 was added at 10 μM final concentration 1 h prior to stimulation of the cells with 100 ng/mL IL-1β or 0.5 M sorbitol. The cells were further grown in DMEM supplemented with 10% FBS for 20 min. Then, the cells were rinsed once with PBS and harvested in 1 × SDS sample buffer. The proteins were separated by 12% SDS-polyacrylamide gel electrophoresis followed by immunoblotting with anti-FLAG M2 antibody for FLAG tag, anti-phospho-p38 MAPK (T180/Y182) antibody (D3F9) for phosphorylated p38s, or anti-MAPKAPK-2 antibody for MK2.

For the analyses with HEK293T cells, 1 × 10^5^ cells were seeded into each well of a 48-well plate. The next day, 10 μM SU derivative or SB202190 was added 1 h prior to stimulation of the cells with 0.2 M NaCl. The cells were further grown in DMEM supplemented with 10% FBS for 20 min. Then, the cells were rinsed once with PBS and harvested in 1 × SDS sample buffer. The proteins were separated by 12% SDS-polyacrylamide gel electrophoresis followed by immunoblotting with anti-phospho-p38 MAPK (T180/Y182) antibody (D3F9) for phosphorylated p38s or anti-p38α antibody for p38α.

## Additional Information

**How to cite this article**: Kondoh, Y. *et al*. Comparative chemical array screening for p38γ/δ MAPK inhibitors using a single gatekeeper residue difference between p38α/β and p38γ/δ. *Sci. Rep.* **6**, 29881; doi: 10.1038/srep29881 (2016).

## Supplementary Material

Supplementary Information
