# Supplementary material for: Comparative chemical array screening for p38γ/δ MAPK inhibitors using a single gatekeeper residue difference between p38α/β and p38γ/δ
Source: Sci Rep. 2016 Jul 19;6:29881. doi: 10.1038/srep29881 (PMC4949465; doi:10.1038/srep29881)
Supplement: Supplementary Information [file srep29881-s1.pdf]

Comparative chemical array screening for p38γ/δ MAPK inhibitors using a single gatekeeper residue difference between p38α/β and p38γ/δ

Authors: Yasumitsu Kondoh[1,2], Kaori Honda[3], Sayoko Hiranuma[2], Teruo Hayashi[1], Takeshi Shimizu[2], Nobumoto Watanabe[3], Hiroyuki Osada[1,2,*]

[1]Antibiotics Laboratory, RIKEN, Saitama 351-0198, Japan

[2]Chemical Biology Research Group, RIKEN Center for Sustainable Resource Science, Saitama 351-0198, Japan

[3]Bio-Active Compounds Discovery Research Unit, RIKEN Center for Sustainable Resource Science, Saitama 351-0198, Japan.

[*]Corresponding author: Hiroyuki Osada, Chemical Biology Research Group, RIKEN Center for Sustainable Resource Science, 2-1 Hirosawa, Wako, Saitama 351-0198, Japan, E-mail: hisyo@riken.jp.

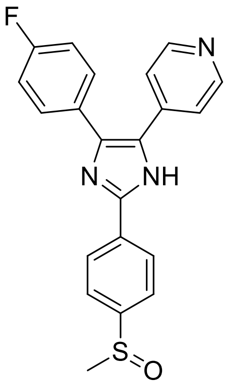

SB203580

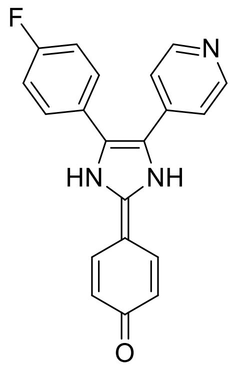

SB202190

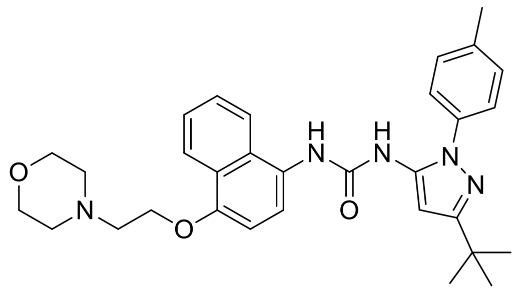

BIRB796

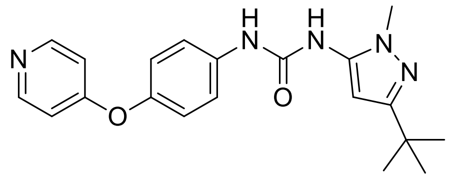

BIRB796 analog
(CHEMBL472620)

Supplementary Figure 1. Chemical structures of SB203580, SB202190, BIRB796 and BIRB796 analog.

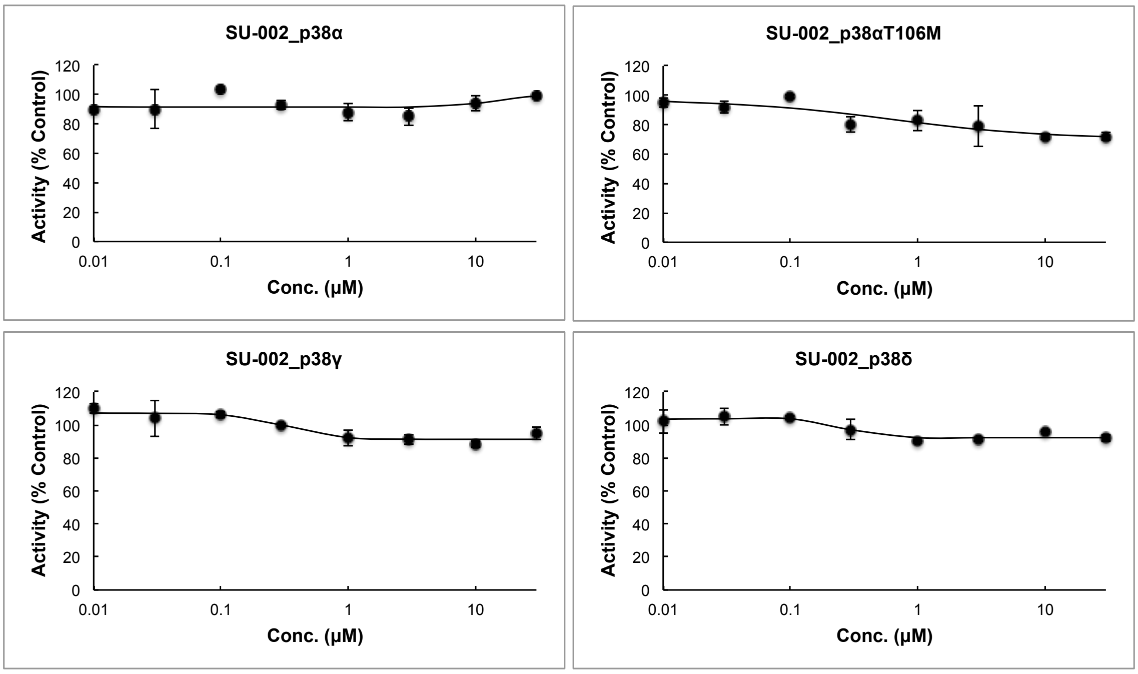

Supplementary Figure 2. Concentration-response curves of four pre-activated p38 MAPK isoforms by SU-002. Results are presented as %kinase activity relative to that in control incubations where compound was omitted (means of duplicate determinations, N=2). Error bar is SD. ATP concentration was Km value (90 μM in p38α; 70 μM in p38αT106M; 15 μM in p38γ and p38δ).

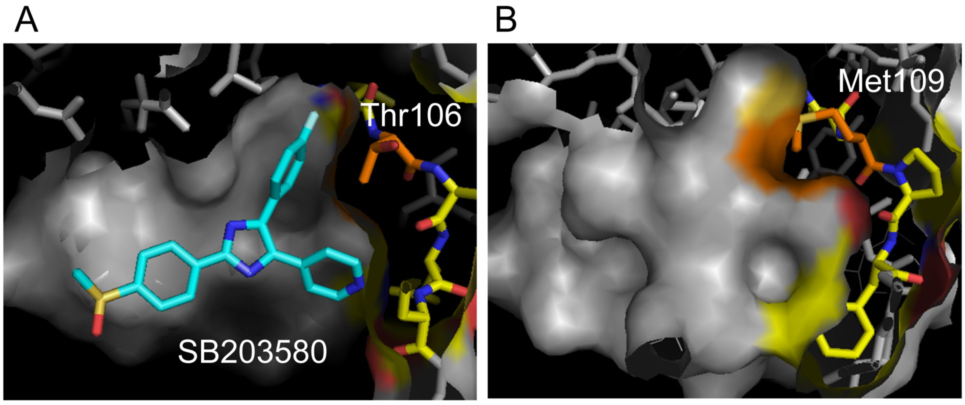

Supplementary Figure 3. Structures of the ATP-binding pockets of p38MAPKs. (A) p38α in complex with SB203580 (from PDB entry 1A9U) (22). (B) p38γ (from PDB entry 1CM8) (23).

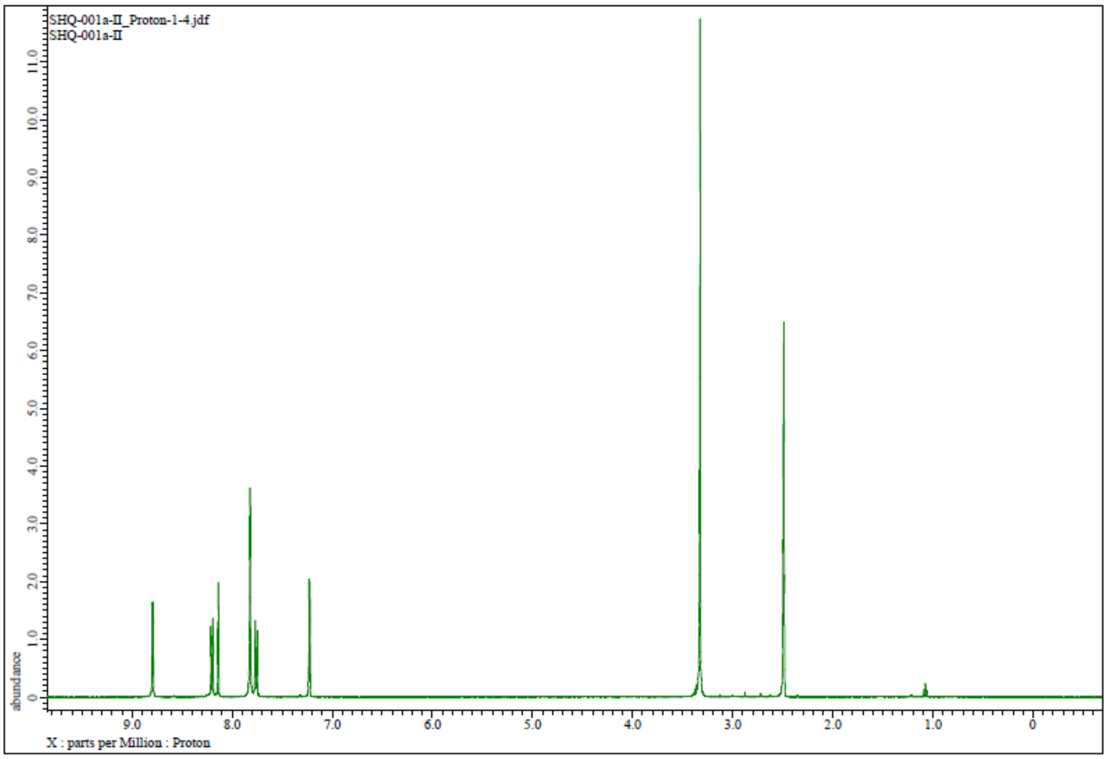

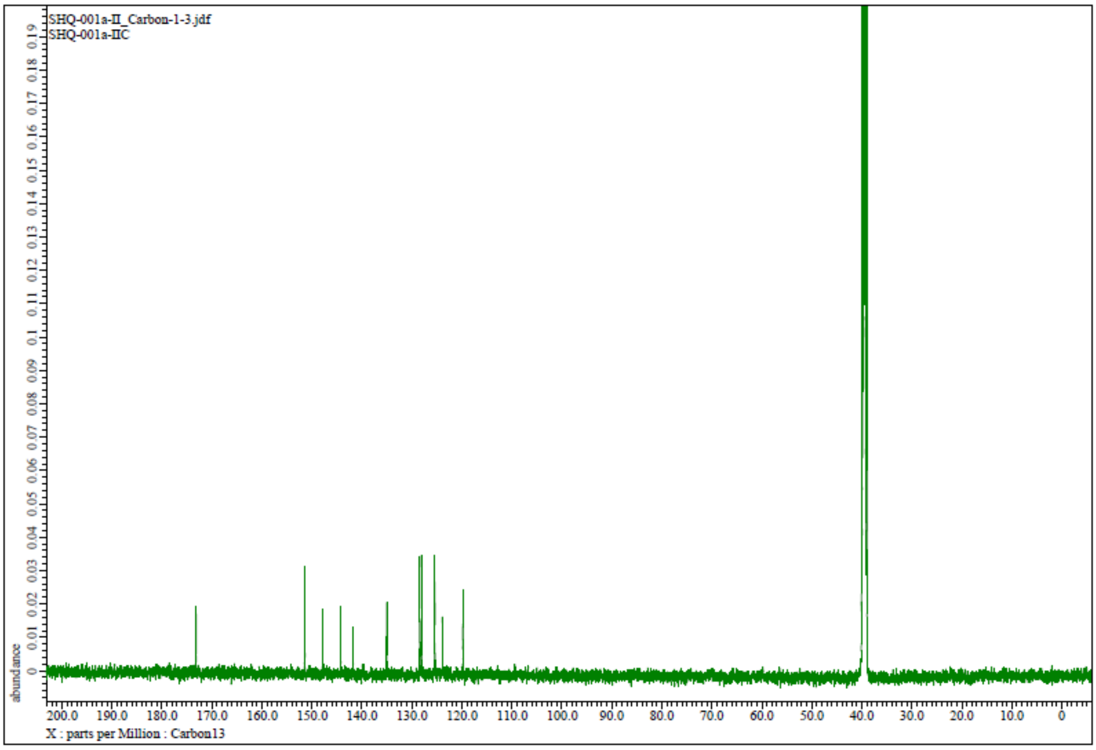

Supplementary Figure 4. ^{1}H NMR and ^{13}C NMR spectra of SU-001.

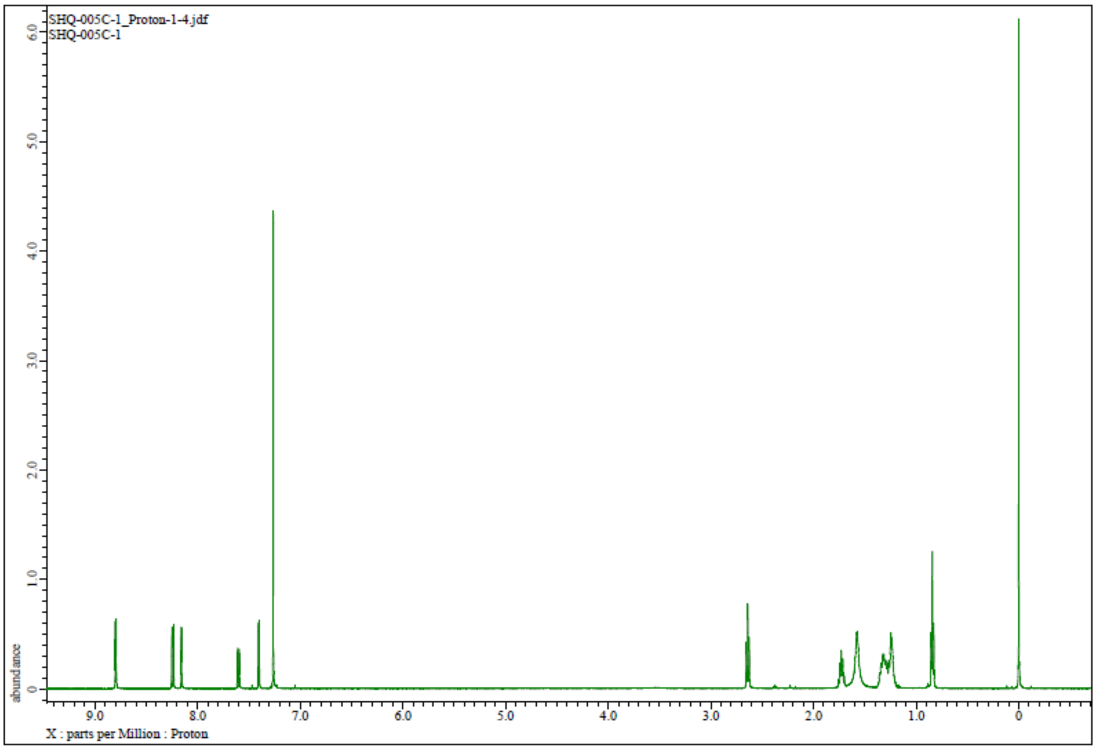

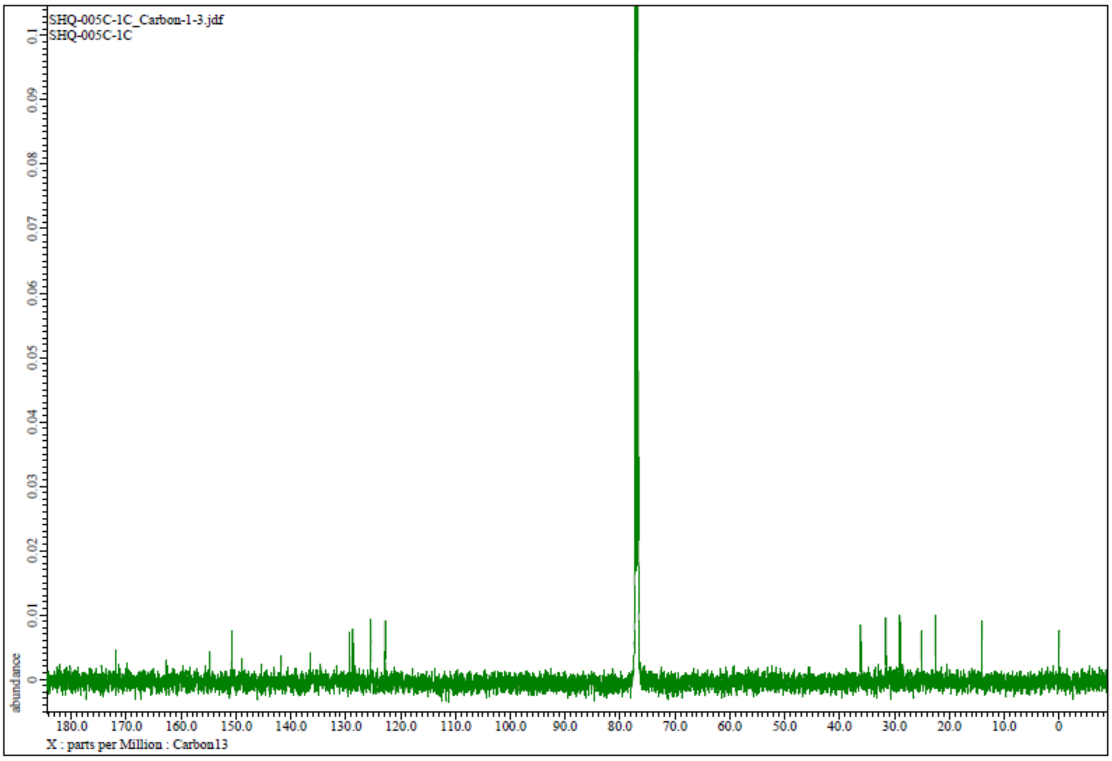

Supplementary Figure 5. ^{1}H NMR and ^{13}C NMR spectra of SU-002.

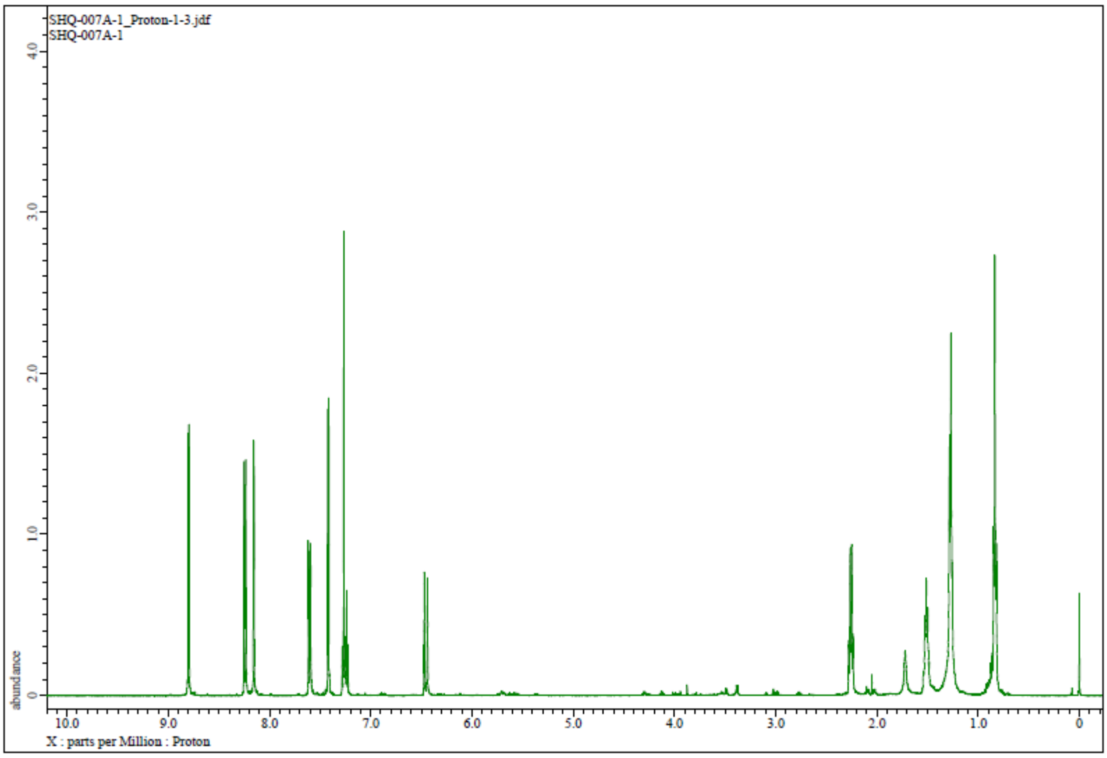

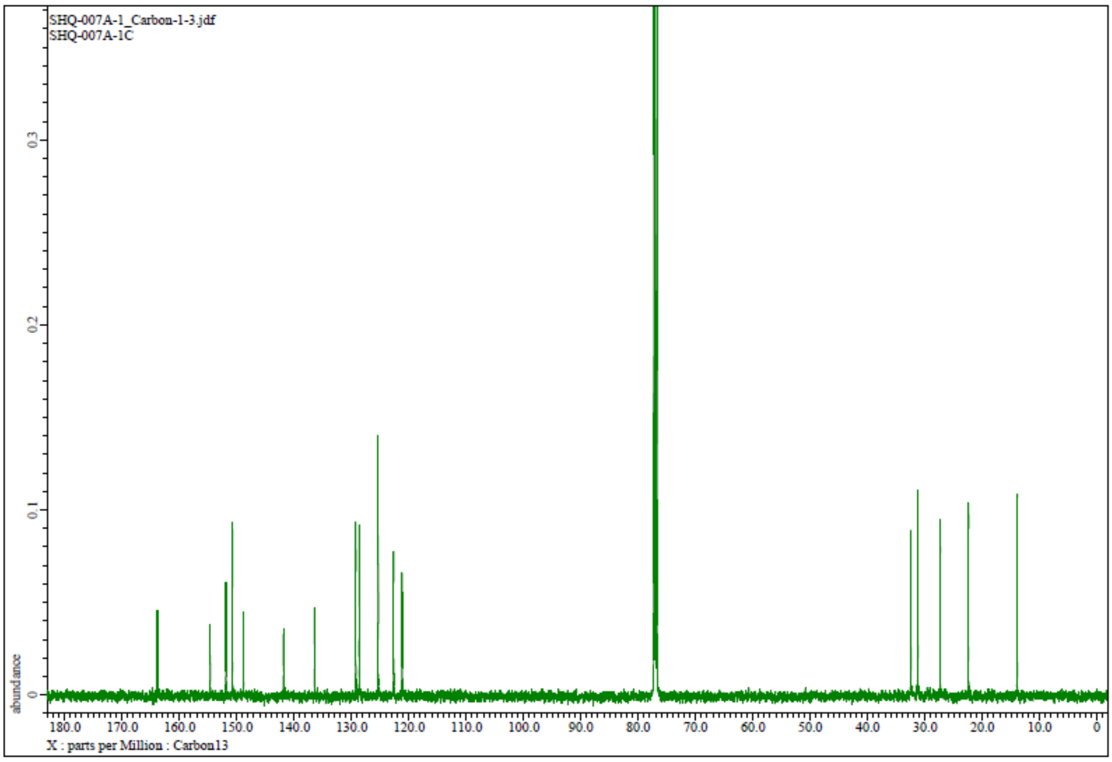

Supplementary Figure 6. ^{1}H NMR and ^{13}C NMR spectra of SU-005.

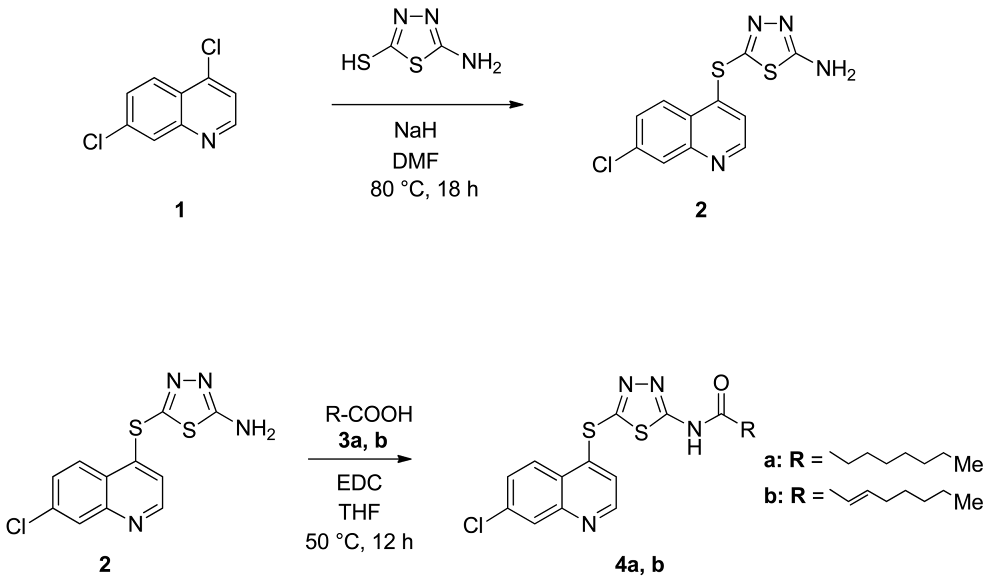

Supplementary Scheme 1. Synthesis schemes of **4a** (SU-002) and **4b** (SU-005).

Table S1. I scores of SB203580 and DMSO spots probed with GST-p38α or GST-p38αT106M.

Array	Block	Row	Column	Name	ΔI score	$I_{p38\alpha}$ score	I_{T106M} score
1	6	1	8	SB203580	13.92	15.17	0.81
1	6	1	9	SB203580	12.74	14.08	0.88
1	30	1	8	SB203580	11.50	11.65	−0.01
1	30	1	9	SB203580	10.46	10.60	0.00
1	1–48	1	10,11	DMSO	−0.28 ± 0.17	−0.07 ± 0.10	0.16 ± 0.11

ΔI was calculated by subtracting I_{T106M} from $I_{p38\alpha}$ ($\Delta I = I_{p38\alpha} - I_{T106M}$). The ΔI score of each spot was calculated by the following formula: ΔI score = $(\Delta I - \Delta I_{mean})/\Delta I_{SD}$. The I score of each spot was calculated by the following formula: I score = $(I - I_{mean})/I_{SD}$. Scores for DMSO spots were the means ± SD (N = 96).

Table S2. I scores of spots for hit compounds binding to GST-p38αT106M specifically.

No.	Array	Block	Row	Column	Name	ΔI score	I_{T106M} score	$I_{p38\alpha}$ score
1	1	7	5	3	SU-002	6.30	4.80	−0.09
1	1	31	5	3	SU-002	4.89	3.66	−0.16
2	1	3	6	2	HTS 09497	3.66	3.15	0.43
2	1	27	6	2	HTS 09497	3.51	2.87	0.20
3	2	9	10	1	NPD2545	2.65	2.27	−0.17
3	2	33	10	1	NPD2545	2.70	2.32	−0.15
4	3	23	6	3	NPD15082	3.65	2.16	−0.48
4	3	47	6	3	NPD15082	4.38	2.82	−0.11

ΔI was calculated by subtracting $I_{p38\alpha}$ from I_{T106M} ($\Delta I = I_{T106M} - I_{p38\alpha}$). The ΔI score of each spot was calculated by the following formula: ΔI score = $(\Delta I - \Delta I_{mean})/\Delta I_{SD}$. The I score of each spot was calculated by the following formula: I score = $(I - I_{mean})/I_{SD}$.

Table S3. Inhibition of p38s by SU-002 and its derivatives.

Compound	Conc.	p38α	p38αT106M	p38γ	p38δ
SU-001	1 μM	91 ± 13	99 ± 0	103 ± 8	94 ± 2
	10 μM	72 ± 2	91 ± 6	95 ± 2	92 ± 1
SU-002	1 μM	88 ± 6	83 ± 7	92 ± 5	90 ± 0
	10 μM	94 ± 5	72 ± 2	88 ± 2	96 ± 0
SU-003	1 μM	92 ± 8	96 ± 4	112 ± 3	99 ± 2
	10 μM	94 ± 6	94 ± 1	104 ± 6	81 ± 1
SU-004	1 μM	91 ± 2	99 ± 0	111 ± 4	100 ± 4
	10 μM	86 ± 3	94 ± 3	104 ± 8	97 ± 3
SU-005	1 μM	91 ± 3	14 ± 4	90 ± 3	85 ± 9
	10 μM	82 ± 4	8 ± 0	80 ± 2	75 ± 2
SU-006	1 μM	86 ± 3	99 ± 4	92 ± 1	96 ± 1
	10 μM	89 ± 3	96 ± 2	103 ± 2	99 ± 8

Results are presented as %kinase activity relative to that in control incubations where compound was omitted (means of duplicate determinations). ATP concentration was Km value (90 μM in p38α; 70 μM in p38αT106M; 15 μM in p38γ and p38δ).
